# IL-15 Overcomes Hepatocellular Carcinoma-Induced NK Cell Dysfunction

**DOI:** 10.3389/fimmu.2018.01009

**Published:** 2018-05-09

**Authors:** Nicholas J. W. Easom, Kerstin A. Stegmann, Leo Swadling, Laura J. Pallett, Alice R. Burton, Dennis Odera, Nathalie Schmidt, Wei-Chen Huang, Giuseppe Fusai, Brian Davidson, Mala K. Maini

**Affiliations:** ^1^Division of Infection and Immunity, Institute of Immunity and Transplantation, University College London, London, United Kingdom; ^2^Tri-Service General Hospital, National Defense Medical Center, Taipei, Taiwan; ^3^Centre for Digestive Diseases, Institute of Liver and Digestive Health, University College London, London, United Kingdom

**Keywords:** NK cells, tissue-resident NK cells, hepatocellular carcinoma, liver tumors, NKG2D, tumor escape, immunotherapy, IL-15

## Abstract

NK cells have potent antitumor capacity. They are enriched in the human liver, with a large subset specialized for tissue-residence. The potential for liver-resident versus liver-infiltrating NK cells to populate, and exert antitumor functions in, human liver tumors has not been studied. We examined liver-resident and liver-infiltrating NK cells directly *ex vivo* from human hepatocellular carcinomas (HCCs) and liver colorectal (CRC) metastases, compared with matched uninvolved liver tissue. We found that NK cells were highly prevalent in both HCC and liver CRC metastases, although at lower frequencies than unaffected liver. Up to 79% of intratumoral NK cells had the CXCR6^+^CD69^+^ liver-resident phenotype. Direct *ex vivo* staining showed that liver-resident NK cells had increased NKG2D expression compared to their non-resident counterparts, but both subsets had NKG2D downregulation within liver tumors compared to uninvolved liver. Proliferation of intratumoral NK cells (identified by Ki67) was selectively impaired in those with the most marked NKG2D downregulation. Human liver tumor NK cells were functionally impaired, with reduced capacity for cytotoxicity and production of cytokines, even when compared to the hypo-functional tissue-resident NK cells in unaffected liver. Coculture of human liver NK cells with the human hepatoma cell line PLC/PRF/5, or with autologous HCC, recapitulated the defects observed in NK cells extracted from tumors, with downmodulation of NKG2D, cytokine production, and target cell cytotoxicity. Transwells and conditioned media confirmed a requirement for cell contact with PLC/PRF/5 to impose NK cell inhibition. IL-15 was able to recover antitumor functionality in NK cells inhibited by *in vitro* exposure to HCC cell lines or extracted directly from HCC. In summary, our data suggest that the impaired antitumor function of local NK cells reflects a combination of the tolerogenic features inherent to liver-resident NK cells together with additional contact-dependent inhibition imposed by HCC itself. The demonstration that IL-15 can recover hepatic NK cell function following tumor exposure supports its inclusion in immunotherapy strategies.

## Introduction

Hepatocellular carcinoma (HCC) remains a very difficult to treat tumor with an exceptionally poor prognosis. It is the second leading cause of cancer deaths worldwide and the fastest increasing cause of cancer-related mortality in Europe (1). The liver is also a common site for metastases from colorectal cancer (CRC); liver metastases carry a very poor prognosis, being typically resistant to chemotherapy and even to early immunotherapy trials with checkpoint inhibitors (2). New approaches able to harness local antitumor immunity are therefore urgently needed for both primary and secondary liver tumors. These require an understanding of the constraints on protective immunity inherent to the liver environment that might be co-opted by tumors in this organ.

Most research into tumor immunology, including in the setting of the liver, has focused on understanding, and attempting to reverse, defects in CD8 T cells (3). However, NK cells also have potent capacity to recognize and kill tumor cells (4, 5). This is evidenced by the multiple NK cell evasion mechanisms exhibited by tumors. NK cell functionality is tuned by the dynamic balance of signals it receives from its environment through a complex array of activatory and inhibitory receptors. One of the major pathways through which NK cells provide immunosurveillance of tumors is through recognition of DNA damage response-induced NKG2D-ligands (NKG2D-L) by the activatory receptor, NKG2D (6, 7). However, the NKG2D pathway plays a complex role in tumor immunity, with some evidence it can also mediate the opposing effect of downmodulating NK cell tumor immunity or even promoting HCC progression (8, 9). Some studies suggested that shedding of tumor NKG2D ligands serves as an escape mechanism by rendering the tumor invisible to NK cells through this receptor and/or by inhibitory effects of the soluble ligands (10–12). Other murine studies have demonstrated that repetitive engagement of NKG2D by its ligands on tumors (13), tumor-associated infiltrates (14), or endothelial cells within tumor vasculature (15) can drive downregulation of this receptor, with consequent NK cell desensitization.

NK cells are a major component of the immunological landscape within human liver, constituting 30–40% of all intrahepatic lymphocytes (16, 17). We and others have recently discovered that a variable fraction (up to 80%) of intrahepatic NK cells are liver-resident, characterized by expression of CXCR6 and CD69, with a distinct transcriptional and functional signature (18–20). These tissue-resident NK cells survive long term in the human liver and are unable to recirculate, while the remaining liver-infiltrating fraction have the transcriptional profile of peripheral NK cells (20, 21). Tissue-resident lymphocytes are adapted for frontline defense, being well positioned to sense and respond immediately to damaged or transformed epithelial cells (22). In the case of tissue-resident T cells, recent work shows they can infiltrate into lung tumors, correlating with a good prognosis, suggesting their propensity for efficient pathogen defense can extend to tumor protection (23, 24). Conversely, we have recently reported that liver-resident T and NK cells have some features reflective of the tolerogenic properties of the liver, such as reduced cytotoxicity (20, 25), which could facilitate tumor immune escape. It is therefore timely to examine the NK cell response to liver tumors to see whether it is influenced by tissue-resident NK cells and their specialized adaptations.

Here, we use direct *ex vivo* analysis of freshly isolated human tissue lymphocytes to compare the contribution of liver-resident and liver-infiltrating NK cells to the composition and functional features of the intratumoral pool. We probe the capacity of HCC to further impair tolerogenic liver NK cells *via* NKG2D downregulation and the potential for cytokine-mediated rescue as an immunotherapeutic strategy in this setting.

## Materials and Methods

### Research Ethics Approval

Blood and tissue sampling was approved by the University College London-Royal Free Hospital Research Ethics Committee, ref nos. 11/WA/0077 (liver explants/resections), 11/H0720/4 (liver perfusates). Blood sampling from healthy donors was approved by the South East Coast Research Ethics Committee, ref no. 11/LO/0421.

### Patient Cohort

Study participants with HCC had the following underlying liver diseases: five hepatitis B virus monoinfection, one hepatitis B/HIV coinfection, two hepatitis C virus monoinfection, one non-alcoholic steatohepatitis, and one autoimmune hepatitis.

### PBMC Isolation and Storage

PBMC were isolated by density gradient centrifugation using Ficoll-Hypaque (GE Healthcare). Cells were either used immediately or counted using trypan blue (Sigma) and transferred to freezing medium (FBS) (Sigma) with 10% dimethylsulfoxide (DMSO) (Sigma) for cryopreservation, initially at −80°C before transfer to liquid nitrogen for long-term storage.

### Intrahepatic Lymphocyte Isolation From Liver Tissue

Single cell suspensions from surgical resection of liver and tumor tissues were generated by enzymatic digestion and mechanical dissociation as previously described (20). In brief, tissues removed from the resected specimen immediately following liver surgery were cut into small pieces and incubated for 30 min at 37°C in HBSS buffer containing 0.0001% DNAse (Roche) and 0.01% collagenase (ThermoFisher). Samples were transferred to C-tubes (Miltenyi Biotec) and processed by gentleMACS (Miltenyi Biotec) using the liver program. Tissue and supernatants were filtered by 70-µm filter and centrifuged at 500 rpm to knockdown large hepatocyte clumps. The supernatant was centrifuged and the pellet resuspended in 30% percoll (GE Healthcare) before further centrifugation at 2,000 rpm for 10 min. The pellet was resuspended in HBSS and layered onto Ficoll-Hypaque as for peripheral blood separation. The lymphocyte layer was removed and cells counted by ADAM counter (NanoEntek) and used immediately.

### Intrahepatic Lymphocyte Isolation From Liver Perfusates

Organ transport and perfusion fluid collected at time of liver transplant was centrifuged in 50 ml falcon tubes (Sarstedt) at 1,800 rpm for 15 min and the supernatant discarded. The tubes were vortexed to disrupt the pellet and the cells pooled and resuspended in RPMI before density gradient centrifugation over Ficoll Hypaque as above.

### Flow Cytometric Staining

All samples were treated with Fc receptor blocking reagent (Miltenyi Biotec) before staining. Surface staining was performed in 96-well plates (Sarstedt) in staining buffer of 50% PBS, 50% Brilliant Violet staining buffer (BD). Fixable live/dead stain (Life Technologies) was added to the staining buffer. Antibody staining was conducted for 15 min at 37°C in the dark before washing with PBS. Samples for surface staining only were fixed in Cytofix (BD). Samples for intracellular staining were fixed in cytofix/cytoperm (BD) for 20 min at 4°C in the dark before staining with intracellular antibodies in saponin buffer [PBS + 1% FBS (Sigma) + 0.1% saponin (Sigma)] for 30 min at 4°C in the dark. Samples were then washed once in saponin buffer and once in PBS. Intranuclear staining was performed using FoxP3 staining buffer (BD). Surface staining was as above, then cells were fixed in buffer A for 10 min at room temperature followed by buffer A with 1:50 buffer B for 30 min at room temperature. Samples were washed in PBS and intranuclear staining performed in PBS for 30 min at 4°C in the dark. Samples were acquired on Fortessa X20 (BD) and data analyzed in FlowJo X (TreeStar).

### Image Analysis and Quantitation of NKG2D Internalization

Magnetic bead isolated human liver NK cells were cocultured with PLC/PRF/5 cells as described below. Cells were surface stained with anti CD3 PE-CF594, anti CD56 PE-Cy7, permeablized and stained intracellularly with anti NKG2D APC and then DAPI nuclear stain. Events were acquired on the Imagestream^X^ (Amnis) and data transferred to IDEAS software (Amnis) for analysis. NKG2D internalization was quantified using the built-in “Internalization Wizard” to erode an object mask by four pixels.

### NK Cell Isolation

Untouched NK cells were isolated from PBMC or intrahepatic lymphocytes using magnetic beads for negative selection, according to the manufacturer’s instructions (Miltenyi Biotec), achieving >95% purity and viability.

### NK Cell—PLC/PRF/5 Cocultures

PLC/PRF/5 cells were plated at a density of 50,000 cells/well in 48-well plates (Costar) and incubated in 0.5 ml CRPMI + 8% FBS for 3 days to adhere to the bottom of the well and grow to semi-confluence. Media was changed and isolated NK cells were added at 200,000 per well, centrifuged at 300 rpm for 3 min and incubated at 37°C for 12 h. Recombinant human IL-15 was added to a final concentration of 1 ng/ml (R&D). Blocking reagents for the appropriate conditions were recombinant human NKG2D final concentration 1 µg/ml (Sino Biological), anti-MICA final concentration 1 µg/ml (R&D), anti-TGFβ receptor final concentration 2 µg/ml (R&D), 0.4 µm transwell inserts (Falcon), or 3-day PLC/PRF/5 media mixed 1:1 with fresh CRPMI + 10% FBS were used for contact experiments. Supernatants containing NK cells were removed and washed with PBS containing EDTA (Sigma) and FBS. For some experiments, removed NK cells were resuspended in fresh medium and incubated for 18 h in the presence of IL-2 20 IU/ml (Miltenyi Biotec), IL-12 12.5 pg/ml (R&D), IL-18 5 ng/ml (MBL), IL-15 1 ng/ml (R&D), IFNα 10 U/ml (PBL interferon) or medium alone.

### HCC Tissue Slices

For some experiments, *ex vivo* human HCC tissue was used instead of cell lines. HCC tissue provided by the Tissue Access for Patient Benefit service at the Royal Free Hospital was sampled using a 5 mm punch biopsy (Stiefel) to prepare cores of uniform diameter, from which 1 mm slices were cut. Thin 5 mm circles of HCC tissue were carefully placed in wells of round bottom 96-well microtiter plates and allowed to rest for 4 h in CRPMI + 8% FBS. Magnetic bead isolated NK cells were stained with Cell Trace Violet (Life Technologies) as per the manufacturer’s protocol before addition to the HCC slices as above.

### CD107a Assays

NK cells were mixed with K562 cells at 1:1 effector:target ratio in 200 µl CRPMI + 10% FBS in 96 well, round bottom plates (Sarstedt). Brefeldin to a final concentration of 1 µg/ml (Sigma), anti-CD107a V450 were added and the plate centrifuged at 300 rpm for 3 min before incubation at 37°C for 3 h and staining for flow cytometry as above.

### Cytokine Stimulation

PBMC, intrahepatic, and intratumor lymphocytes were stimulated with 5 ng/ml IL-12 and 50 ng/ml IL-18 for 12 h in the presence of 1 µg/ml brefeldin, before intracellular cytokine staining as above.

### Statistical Analysis

The Mann–Whitney *U* test was used for comparisons of two unpaired groups where *n* = 6 or greater, unpaired *t* test with Welch’s correction was used for comparisons of smaller groups. The Wilcoxon signed rank test was used for comparisons of two paired groups. Spearman rank test was used for correlations of continuous variables. These tests were performed in GraphPad Prism version 6. MANOVA was performed in SPSS (IBM) version 24. *p* ≤ 0.05 was considered to be significant for all tests. Figures are labeled: *p ≤ 0.05; **p ≤ 0.005; ***p ≤ 0.001; ****p ≤ 0.0001.

## Results

### Liver Tumors Contain a High Proportion of NK Cells Including a Tissue-Resident Subset

**Figure 1:**
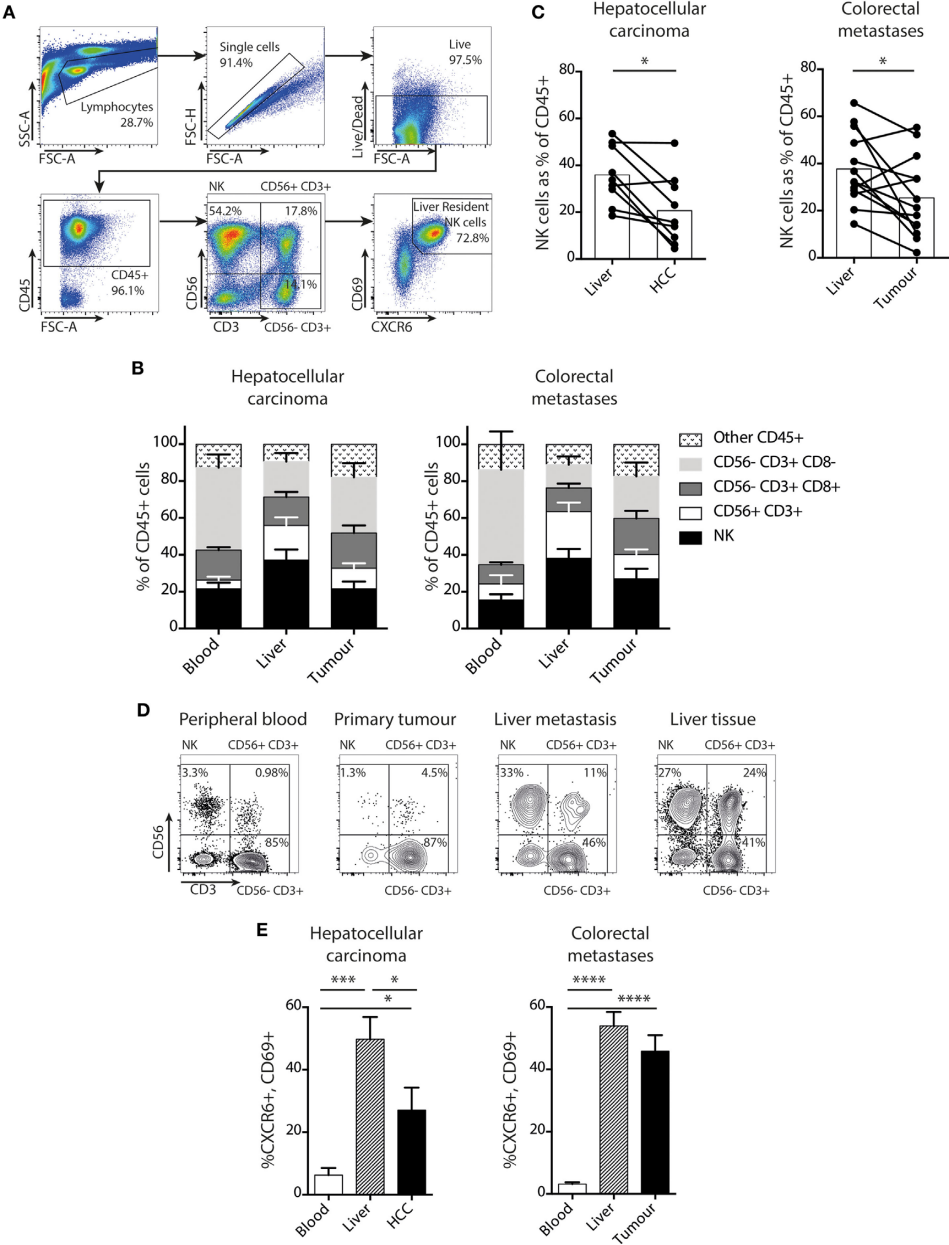
NK cells, including a liver-resident subset, are prevalent in liver tumors. **(A)** Gating strategy for identification of intrahepatic or tumor-infiltrating NK cells by flow cytometry, showing identification of CXCR6^+^CD69^+^ liver-resident NK cells (CD45^+^CD56^+^CD3^−^CD69^+^CXCR6+). **(B)** Proportion of NK cells, CD56^+^CD3^+^ cells, CD56^−^CD8^+^ T cells, and CD56^−^CD8^−^ T cells in blood, liver, and tumor tissue in patients with hepatocellular carcinoma (HCC) (*n* = 6) and CRC (*n* = 10). Bars show mean and SEM, *p* values determined by MANOVA. **(C)** Total NK cells (CD56^+^CD3^−^) as a proportion of CD45^+^ lymphocytes in HCC (*n* = 9) and CRC (*n* = 13) paired liver and tumor. Bars indicate mean of each group. **(D)** Flow cytometry dot plots showing CD45^+^ lymphocytes divided into NK, CD56^+^ T cells, and CD56^−^ T cells for lymphocytes derived from peripheral blood, primary colonic adenocarcinoma, liver metastasis, and unaffected liver tissue in one individual. **(E)** CXCR6^+^CD69^+^ liver-resident NK cells as a proportion of total NK cells in blood, liver, and tumor from HCC (*n* = 10) and CRC (*n* = 13) patients by flow cytometry. Groups were compared using Mann–Whitney *U* test (unpaired) and Wilcoxon matched-pairs signed rank test (paired analyses). *p* ≤ 0.05 was considered to be significant for all tests. Figures are labeled: *p ≤ 0.05; **p ≤ 0.005; ***p ≤ 0.001; ****p ≤ 0.0001.

In order to determine whether NK cells make up a substantial proportion of the immune infiltrate of liver tumors, we compared their direct *ex vivo* frequency with those of CD8 T cells (CD3^+^CD56^−^CD8^+^), CD8 negative T cells (CD3^+^CD56^−^CD8^−^), and CD56^+^ T cells (CD3^+^CD56^+^) in blood, healthy liver margins, and HCC or CRC liver metastases. Cells were identified by multiparameter flow cytometry of single cell suspensions derived from liver and tumor tissue (gated as shown in Figure 1A). In both primary liver tumors (HCC) and secondary liver tumors arising from CRC metastases NK cells were prevalent, accounting for around 25% of the CD45^+^ lymphocytes, with a frequency intermediate between blood and uninvolved liver. We term NK cells derived from liver tissue or from liver perfusion fluid “intrahepatic NK cells,” and NK cells derived from tumor tissue as “tumor-infiltrating NK cells.” In both settings, NK cell and CD8 T cell proportions were similar, with mean proportions of 23.7 and 19.6% for NK and CD8 T cells in HCC, and 27.2 and 21.0% for NK and CD8 T cells in CRC metastases (Figure 1B). In a paired analysis, NK cells in tumors comprised a slightly lower proportion of the CD45^+^ lymphocyte pool than in unaffected liver tissue (Figure 1C). However, NK cells were detectable in all liver tumor infiltrates and constituted up to a maximum of 49% in HCC and 55% in CRC metastases. In one example of blood, primary colonic adenocarcinoma, liver metastasis, and liver tissue from a single patient, the composition of the CD45^+^ lymphocyte pool in the liver metastasis was more similar to that of the unaffected liver than to the peripheral blood or the primary tumor (Figure 1D). These data suggested that both primary and secondary liver tumors acquire an NK-enriched lymphocyte pool, a prototypic feature of the liver immune landscape.

We next asked whether a recently defined subset of liver-resident NK cells was able to infiltrate liver tumors. These tissue-resident NK cells are marked by surface co-expression of CXCR6 and CD69 and characterized by low cytotoxicity and pro-inflammatory cytokine capacity (20). We found that both HCC and CRC metastases contained a high proportion of NK cells with the liver-resident CXCR6^+^CD69^+^ phenotype, compared to the very low frequencies present in the blood (Figure 1E). Tumor-infiltrating NK cells in HCC contained up to 79% with a liver-resident phenotype, although the mean of 10 HCC was significantly less than in the uninvolved distant liver margins (28 versus 49%, respectively). In the 13 patients with CRC metastases examined, the proportion of NK cells with a liver-resident phenotype was comparable to the healthy distant liver margin (mean 46 versus 54%, respectively, Figure 1E). Thus both HCC and CRC metastases had an NK cell composition that was more reflective of the surrounding liver tissue than peripheral blood, with a similar proportion of liver resident and liver-infiltrating “non-resident” (CXCR6^−^CD69^−^) NK cells to the unaffected liver (Figure 1E). Because liver-resident NK cells have distinct phenotypic and functional features, all subsequent comparisons of NK cells within tumors and surrounding liver were analyzed for both liver-resident and liver-infiltrating subsets.

### Intratumoral NK Cells Have Downregulated NKG2D With Selective Impaired Proliferation of the NKG2D^lo^ Fraction

**Figure 2:**
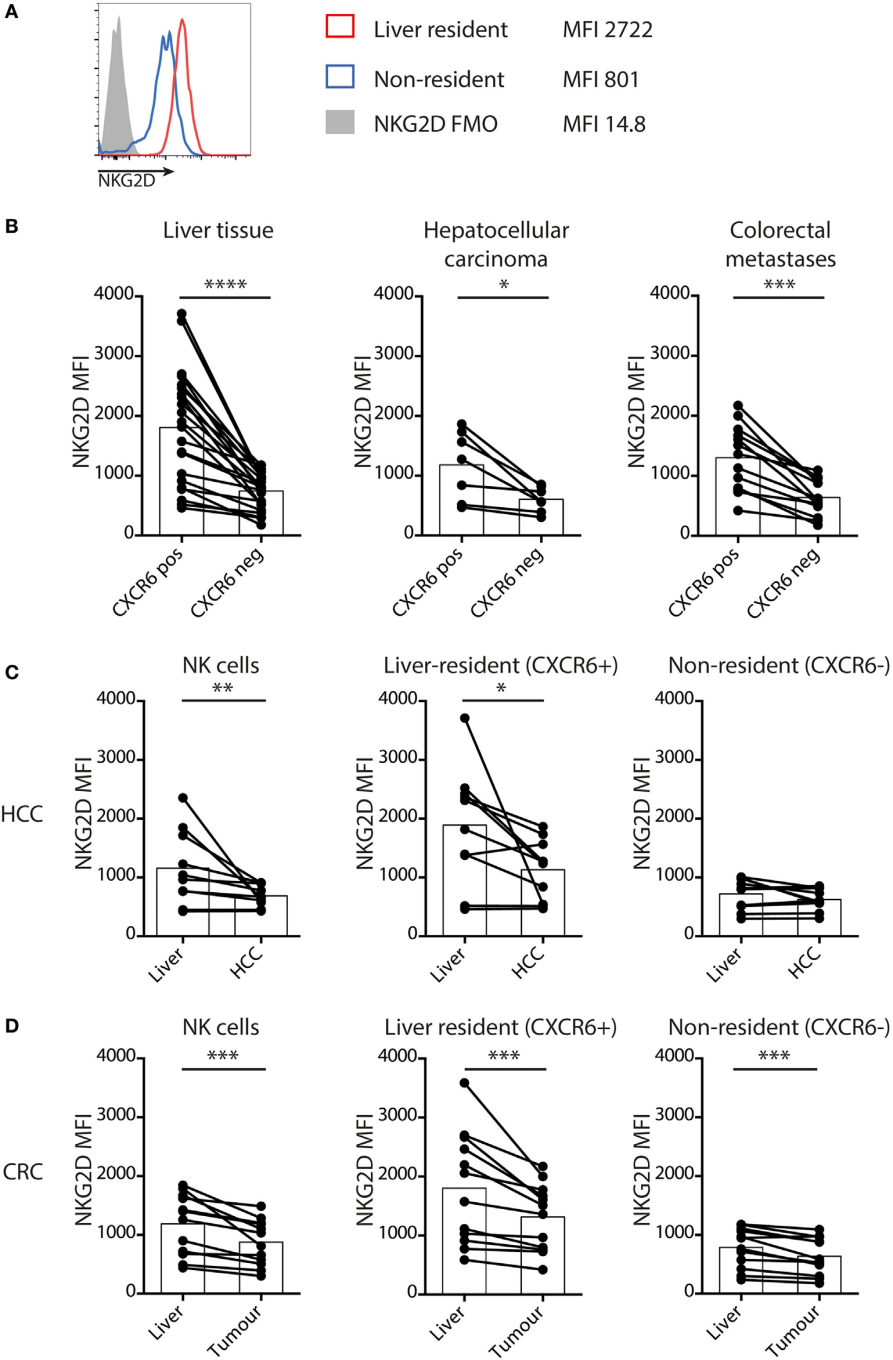
Liver-resident NK cells have increased NKG2D that is downregulated in tumors. **(A)** Representative histograms to show NKG2D expression by liver-resident and non-resident NK cells. **(B)** NKG2D expression by mean fluorescence intensity (MFI) on tissue-resident (CXCR6^+^CD69+) and non-resident (CXCR6^−^CD69^−^) liver (*n* = 23), hepatocellular carcinoma (HCC) (*n* = 10), and CRC (*n* = 13) NK cells quantified by flow cytometry. Bars indicate mean of each group. **(C)** NKG2D expression by MFI on total NK cells, liver-resident (CXCR6^+^CD69+) and non-resident (CXCR6^−^CD69^−^) NK cells in HCC and paired liver (*n* = 10) quantified by flow cytometry. Bars indicate mean of each group. **(D)** NKG2D expression by MFI on total NK cells, liver resident (CXCR6^+^CD69+) and non-resident (CXCR6^−^CD69^−^) NK cells in CRC and paired liver (*n* = 13) quantified by flow cytometry. Bars indicate mean of each group. Groups were compared using Wilcoxon matched-pairs signed rank test. *p* ≤ 0.05 was considered to be significant for all tests. Figures are labeled: *p ≤ 0.05; **p ≤ 0.005; ***p ≤ 0.001; ****p ≤ 0.0001.

We have previously reported that NK cells maintain paradoxically high expression of NKG2D within the liver, even in the presence of NKG2D ligands on neighboring T cells in the context of HBV-related inflammation (26). To further investigate this, we compared NKG2D expression on the CXCR6^+^CD69 liver-resident subset with non-resident, liver-infiltrating NK cells (Figure 2A) in HCC, CRC metastases, and matched uninvolved liver tissue. In every case, the concentration of NKG2D was higher on the CXCR6^+^ liver-resident NK cells than their liver-infiltrating counterparts (Figure 2B).

However, comparison of NK cells within tumors with those in matched liver revealed downregulation of NKG2D on those within HCC (Figure 2C) and CRC metastases (Figure 2D). On the non-resident NK cells from liver containing CRC metastases the NKG2D expression was already low, with a further subtle but consistent decrease within metastases (Figure 2D). Instead, NKG2D downregulation on global intratumoral NK cells was largely attributable to decreases on the liver-resident subset (Figures 2C,D). We postulated this downmodulation of NKG2D expression may result from engagement by NKG2D-L on liver tumors, vasculature or infiltrates, as described in other settings (13–15).

**Figure 3:**
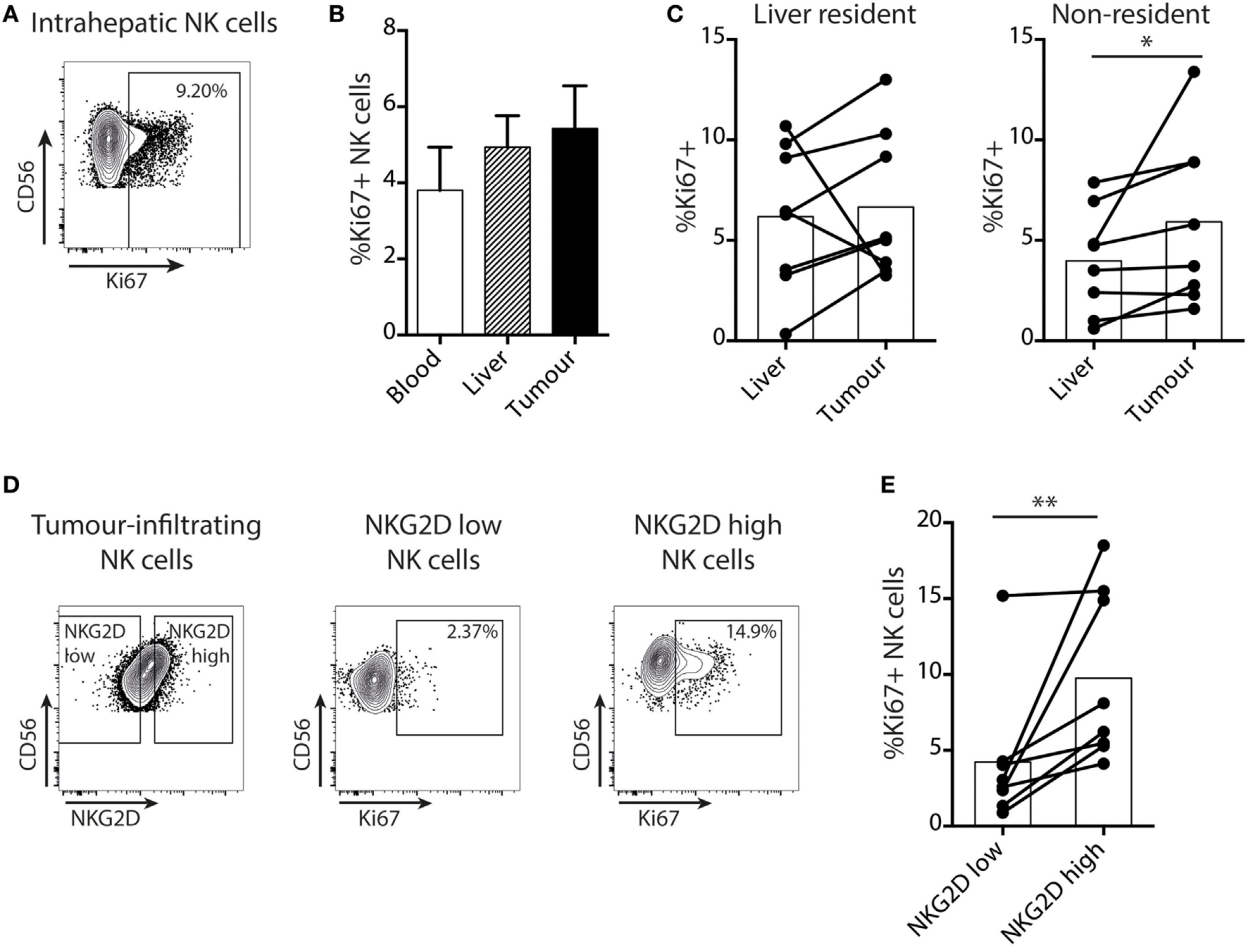
Proliferation of intratumoral NK cells is selectively impaired in the NKG2D^lo^ fraction. **(A)** Representative example of intranuclear Ki67 staining on intrahepatic NK cells by flow cytometry. **(B)** Summary data for Ki67 expression by flow cytometry on total NK cells from paired blood, liver, and tumor. Bars shown mean and SEM. **(C)** Ki67 expression by liver-resident and non-resident NK cells from paired liver and tumor (*n* = 8). Bars indicate mean of each group. **(D)** Example of gating of highest and lowest 25% of tumor-infiltrating NK cells by NKG2D expression, and Ki67 expression in these two groups. **(E)** Ki67 expression on the highest and lowest 25% of tumor-infiltrating NK cells by NKG2D expression (*n* = 8). Bars indicate mean of each group. Ex indicates tissue explant. Groups were compared using Mann–Whitney *U* test (unpaired) and Wilcoxon matched-pairs signed rank test (paired analyses). *p* ≤ 0.05 was considered to be significant for all tests. Figures are labeled: *p ≤ 0.05; **p ≤ 0.005.

Next, we examined NK cell turnover using *ex vivo* staining for the proliferation marker Ki67. Overall NK cell proliferation was maintained within liver tumors compared to surrounding liver tissue and blood (Figures 3A,B). NK cell Ki67 was slightly higher in the tumor-infiltrating (non-resident) fraction than in the distant liver margin (Figure 3C), possibly indicating an initial proliferation on first encountering tumor cells. We then investigated whether the NK cell NKG2D downregulation we observed within tumors affected their proliferative capacity by comparing Ki67 expression on NKG2D^hi^ versus NKG2D^lo^ NK cells, gating on those with the highest and lowest 25% of NKG2D expression, respectively (Figure 3D). Intratumoral NK cells that had downregulated NKG2D had significantly lower proliferation than their NKG2D^hi^ counterparts (Figure 3E).

### Intratumoral NK Cells Have Impaired Cytokine Production and Target Cell Cytotoxicity

**Figure 4:**
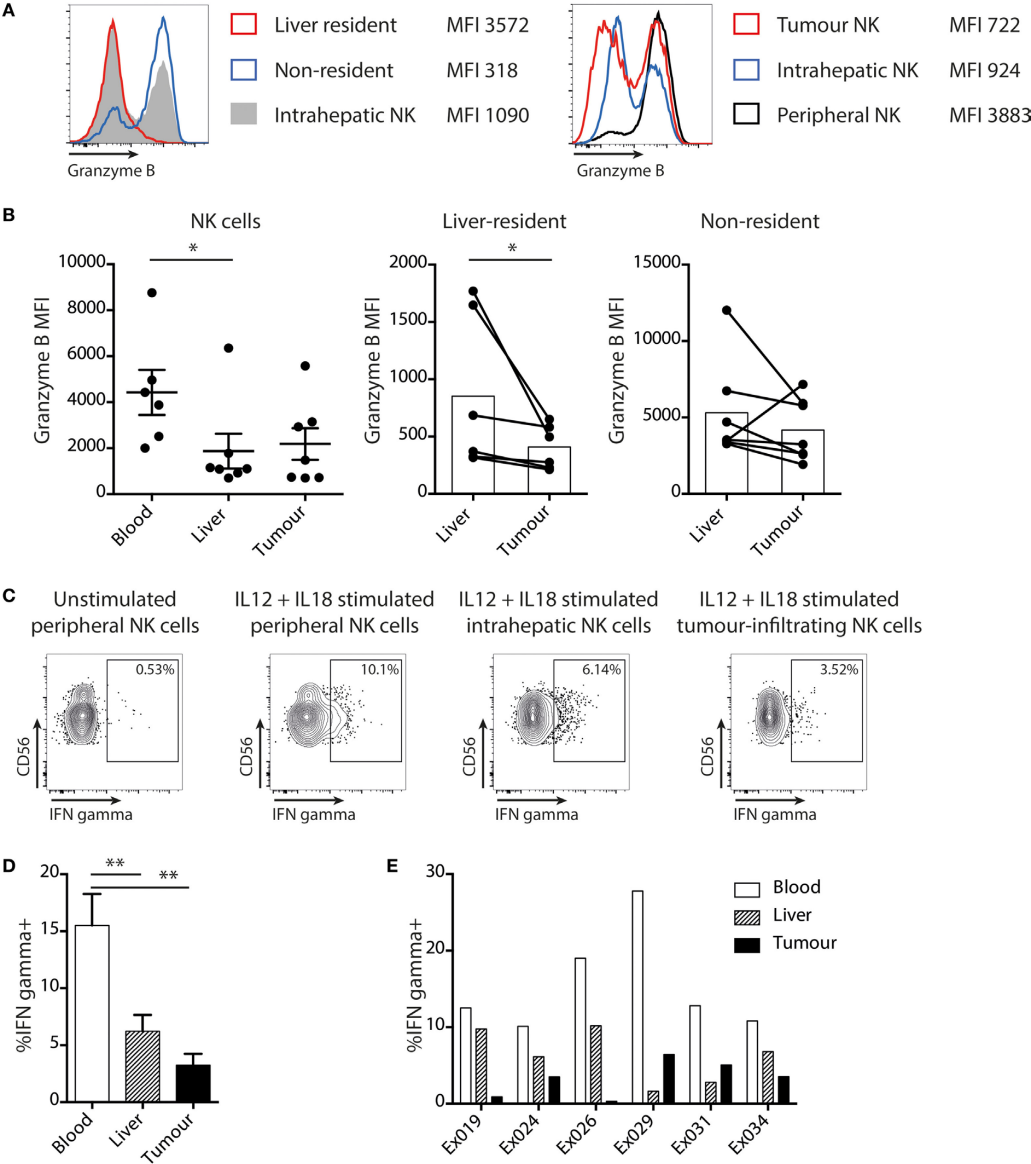
Liver tumor NK cells are functionally impaired. **(A)** Representative histograms of granzyme B staining on total intrahepatic NK cells, liver resident and non-resident subsets of NK cells, and on matched peripheral, liver, and tumor NK cells. **(B)** Granzyme B expression (mean fluorescence intensity) on global (mean and SEM shown), liver-resident and non-resident NK cells in matched peripheral blood, liver, and tumor. Bars indicate mean of each group. **(C)** Representative example of intracellular cytokine staining for IFNγ in unstimulated NK cells and NK cells stimulated with IL12 and IL18. **(D)** Summary data showing IFNγ production by total NK cells from blood, liver, and tumor (bars shown mean and SEM) and **(E)** for individual matched blood and tissue samples. Ex indicates tissue explant. Groups were compared using Mann–Whitney *U* test (unpaired) and Wilcoxon matched-pairs signed rank test (paired analyses). *p* ≤ 0.05 was considered to be significant for all tests. Figures are labeled: *p ≤ 0.05; **p ≤ 0.005.

To further investigate the impact of the liver tumor environment on NK cell antitumor efficacy, we examined their cytotoxicity and cytokine-producing capacity. As previously described (20), the key cytotoxic mediator granzyme B was observed to be markedly reduced in liver-resident (CXCR6^+^CD69+) NK cells and was instead mainly confined to the recirculating non-resident fraction (Figure 4A). Consistent with their enrichment with the liver-resident subset, both intrahepatic and tumor NK cells tended to have reduced granzyme B compared to circulating NK cells (Figures 4A,B). Of note, NK cell cytotoxic potential was further reduced for the resident fraction within tumors compared to distant liver margins (Figure 4B).

NK cells isolated from matched liver tumors, distant margins, and blood were also compared for their capacity to produce IFNγ, another antitumor effector mechanism, following stimulation with IL-12/18 (Figure 4C). A significantly lower proportion of NK cells within tumors and liver were able to produce IFNγ than in the blood (Figure 4D); in four out of six cases, production was even lower within the tumor than the uninvolved liver (Figure 4E).

Taken together, these data indicate that local NK cells have NKG2D downregulation with impaired cytolytic and non-cytolytic potential against liver tumors.

### NK Cell Coculture With an HCC Cell Line Recapitulates NKG2D Downregulation and Functional Inhibition in a Contact-Dependent Manner

**Figure 5:**
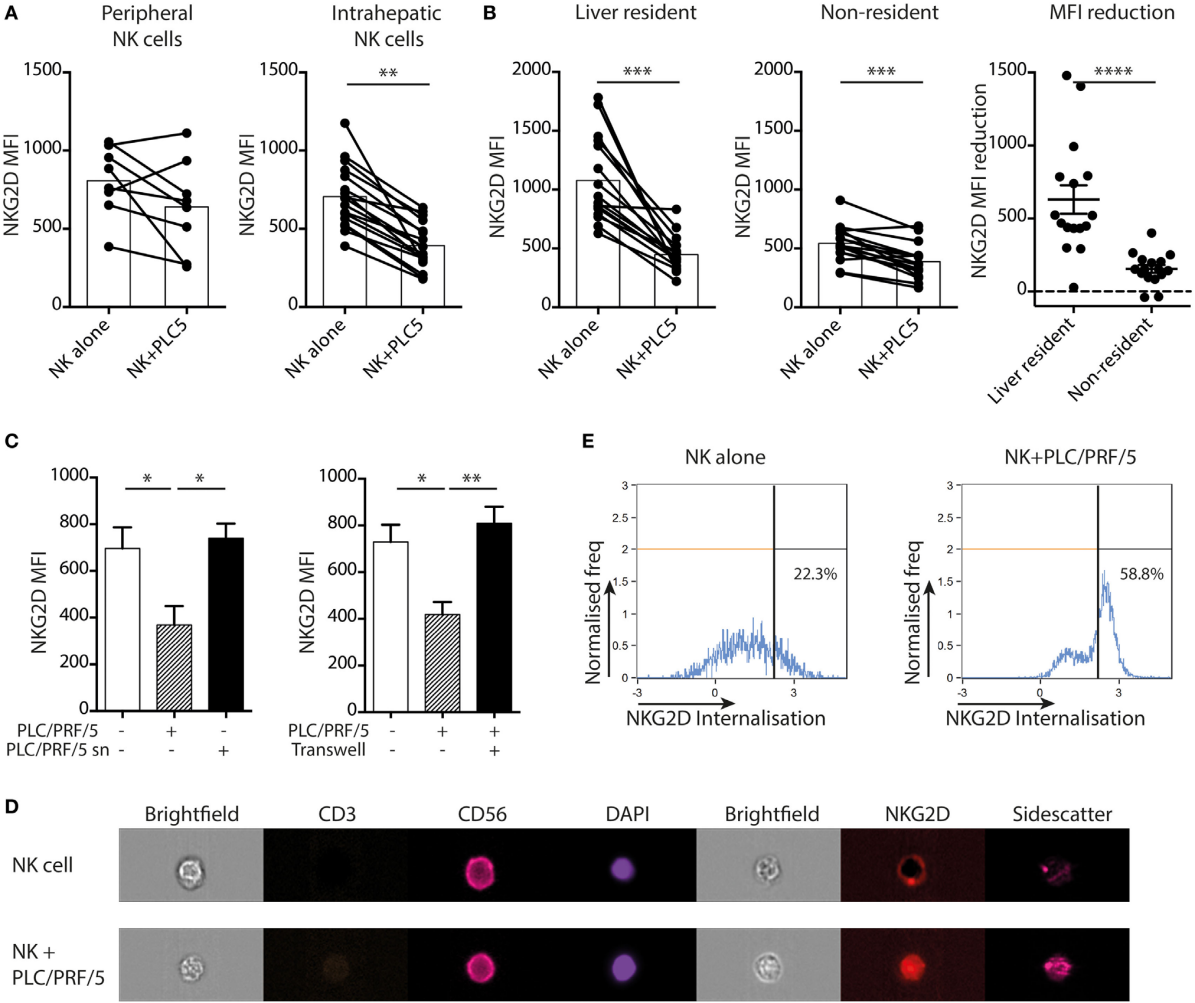
PLC/PRF/5 cells induce NKG2D downregulation on intrahepatic NK cells in a cell-contact-dependent manner. **(A)** NKG2D expression [mean fluorescence intensity (MFI)] by peripheral (*n* = 8) and intrahepatic (*n* = 17) NK cells following 18 h coculture with or without PLC/PRF/5 cells. Bars indicate mean of each group. **(B)** NKG2D expression (MFI) by liver-resident and non-resident intrahepatic NK cells following 12 h coculture with or without PLC/PRF/5 cells (*n* = 16), bars indicate mean of each group, and comparison of amount of reduction in NKG2D MFI for liver-resident and non-resident NK cells after PLC/PRF/5 coculture, mean and SEM shown. **(C)** NKG2D expression by intrahepatic NK cells following 12 h coculture alone or with PLC/PRF/5 cells or with their culture supernatant (sn) diluted 1:1 with fresh media (*n* = 4) or separated by a transwell (*n* = 4). **(D)** Representative example showing NKG2D internalization in NK cells following 12 h coculture with PLC/PRF/5 cells by imaging cytometry. **(E)** Summary data showing NKG2D internalization score (computed by IDEAS software, described in Section “Materials and Methods”) following 12 h coculture with or without PLC/PRF/5 cells. Groups were compared using Mann–Whitney *U* test (unpaired) and Wilcoxon matched-pairs signed rank test except **(C)**, unpaired *t*-test with Welch’s correction. *p* ≤ 0.05 was considered to be significant for all tests. Figures are labeled: *p ≤ 0.05; **p ≤ 0.005; ***p ≤ 0.001; ****p ≤ 0.0001.

To investigate whether HCC itself, instead of its tumor milieu, is capable of imposing the changes observed on *ex vivo* tumor NK cells, we used a coculture system. To mimic the interaction between NK cells and HCC we used PLC/PRF/5 cells, an adherent line derived from HCC that expresses multiple ligands for NK cell receptors, including HLA class I and the NKG2D ligand MICA (Figure S1 in Supplementary Material). Overall, tumor-infiltrating NK cells resembled hepatic NK cells much more closely than peripheral NK cells, so liver NK cells were used to examine the functional impairment imposed on tumor-infiltrating NK cells. Magnetic beads were used to isolate liver NK cells by negative selection and these were then cocultured overnight with or without the HCC cell line. Intrahepatic NK cells downregulated NKG2D on their cell surface on coculture with PLC/PRF/5, an effect not seen consistently with peripheral NK cells (Figure 5A). This downregulation was consistently seen on both liver-resident and non-resident fractions. However, as previously observed directly *ex vivo*, the degree of downmodulation was more striking on the liver-resident subset that had higher baseline NKG2D staining (Figure 5B).

We next questioned whether NKG2D downregulation required cell contact or was mediated by a soluble factor produced by HCC. We observed that NKG2D downregulation did not occur when intrahepatic NK cells were cultured in 50% PLC/PRF/5 culture supernatant, suggesting it was not mediated by a soluble factor (Figure 5C). In addition, this HCC line was no longer able to induce NKG2D downregulation once it was separated from NK cells by a semipermeable membrane in a transwell system (Figure 5C), confirming the requirement for contact. To investigate the fate of cell surface NKG2D, we used imaging cytometry to visualize and quantitate the cellular localization of NKG2D molecules. Whereas CD56 showed a ring-like cell membrane-associated distribution, NKG2D was visualized within the NK cell cytoplasm following engagement with PLC/PRF/5 (representative example and summary data quantitating internalization following NK cell culture overnight with or without PLC/PRF/5, Figures 5D,E). Thus, the interaction of NKG2D-L on PLC/PRF/5 with NKG2D on intrahepatic NK cells drove NKG2D internalization, accounting for the downregulation observed.

**Figure 6:**
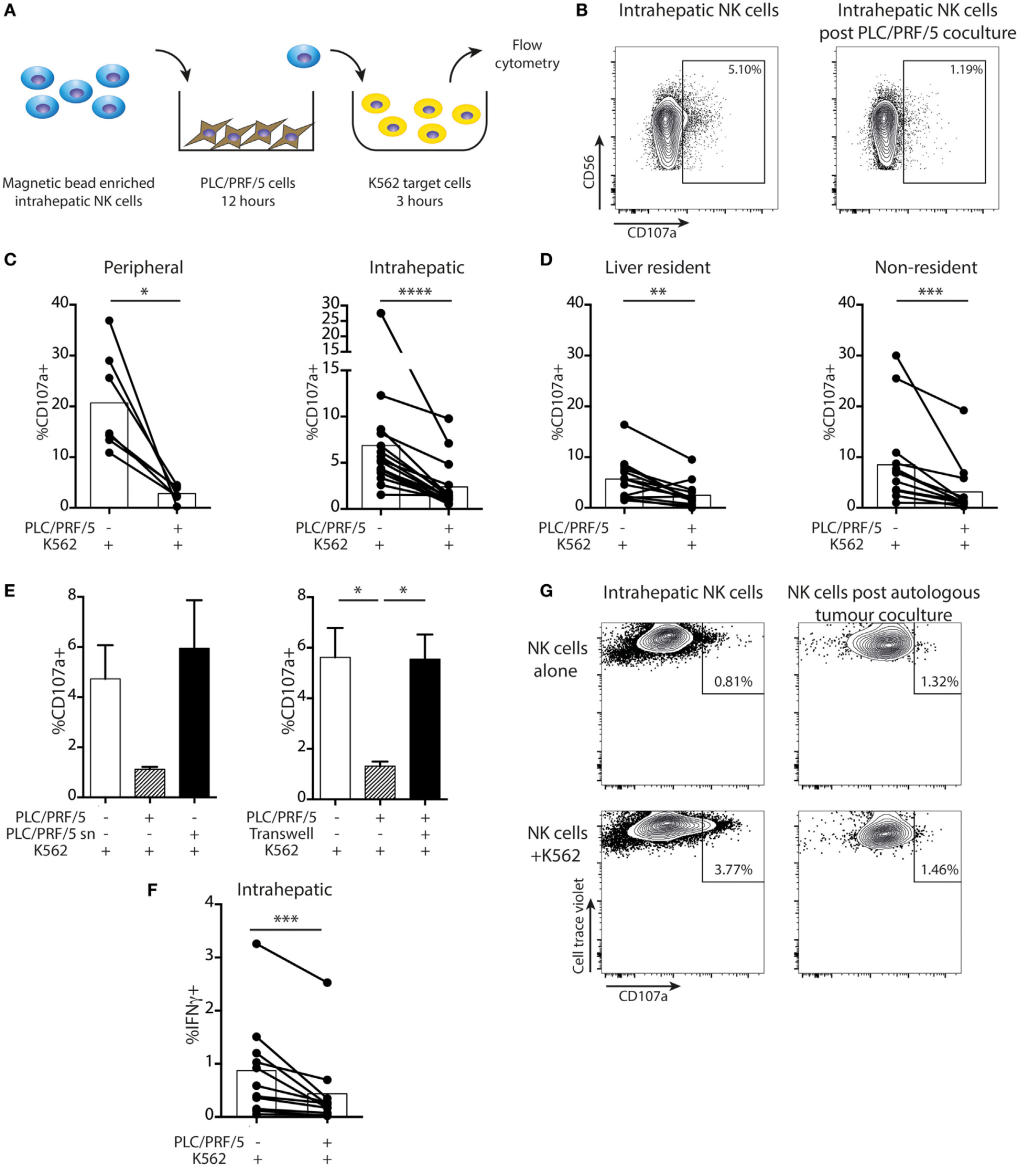
Intrahepatic NK cells are functionally impaired upon exposure to PLC/PRF/5 cells. **(A)** Diagram showing experimental design of PLC/PRF/5 coculture followed by K562 challenge. **(B)** Representative example of CD107a staining measured by flow cytometry on intrahepatic NK cells following 12 h coculture with PLC/PRF/5 cells (or alone) followed by challenge with K562 cells. **(C)** Summary data of CD107a expression by peripheral (*n* = 7) and intrahepatic NK cells (*n* = 16) following K562 challenge after PLC/PRF/5 coculture. **(D)** Summary data of CD107a expression on liver resident and non-resident intrahepatic NK cells (*n* = 14) following K562 challenge after PLC/PRF/5 coculture. **(E)** CD107a staining following K562 challenge after coculture with PLC/PRF/5 cells with culture supernatant diluted 1:1 with fresh media or separated by a transwell. **(F)** Intracellular IFNγ staining in intrahepatic NK cells (*n* = 11) following K562 challenge after PLC/PRF/5 coculture. Bars indicate mean of each group. **(G)** CD107a expression on intrahepatic NK cells following 12 h coculture with or without autologous hepatocellular carcinoma followed by challenge with K562 cells. Cells were gated on cell trace violet-positive NK cells (used to distinguish added intrahepatic NK cells from NK cells present in the autologous tumor). Groups were compared using Mann–Whitney *U* test (unpaired) and Wilcoxon matched-pairs signed rank test (paired analyses) except **(E)**, unpaired *t*-test with Welch’s correction. *p* ≤ 0.05 was considered to be significant for all tests. Figures are labeled: *p ≤ 0.05; **p ≤ 0.005; ***p ≤ 0.001; ****p ≤ 0.0001.

Next, we tested the impact of HCC on intrahepatic NK cell cytotoxicity using a two-stage challenge model (Figure 6A). After coculture with PLC/PRF/5 cells overnight, NK cells were washed off and challenged with K562 cells at 1:1 ratio to assess residual cytotoxicity. Cytotoxic potential, as measured by CD107a expression, was consistently reduced in both peripheral and intrahepatic NK cells following exposure to PLC/PRF/5 (representative example Figure 6B, summary data Figure 6C). This effect was seen in both resident and non-resident NK cell populations, although liver-resident NK cells had a lower level of baseline degranulation (Figure 6D). The HCC-driven impairment in NK cell cytotoxicity was abrogated using PLC/PRF/5 supernatants or upon separation by a transwell insert (Figure 6E), again implying a cell contact-dependent mechanism. IFNγ production was also significantly impaired after coculture with PLC/PRF/5 HCC cells (Figure 6F), with the bulk of the decline seen in non-resident NK cells (Figure S2 in Supplementary Material).

Using recombinant soluble NKG2D or anti-MICA to block the NKG2D-mediated NK cell-PLC/PRF/5 interaction, there was only a non-significant trend toward recovery of NKG2D expression by the NK cells and no effect on cytotoxic function (Figures S3A,B in Supplementary Material). Similarly, there was no restoration of NKG2D expression or NK cell cytotoxicity with anti-TGFβ receptor blockade (Figure S3A,B in Supplementary Material).

In one case, we obtained sufficient material from an HCC to test the impact of the autologous tumor on intrahepatic NK cells. Intrahepatic NK cells rested overnight were capable of some degranulation when exposed to K562 targets. By contrast, NK cells extracted from the same liver sample but exposed to autologous tumor overnight showed no cytotoxicity toward K562 challenge (Figure 6G).

### IL-15 Restores NK Cell Function Following HCC-Driven Inhibition

**Figure 7:**
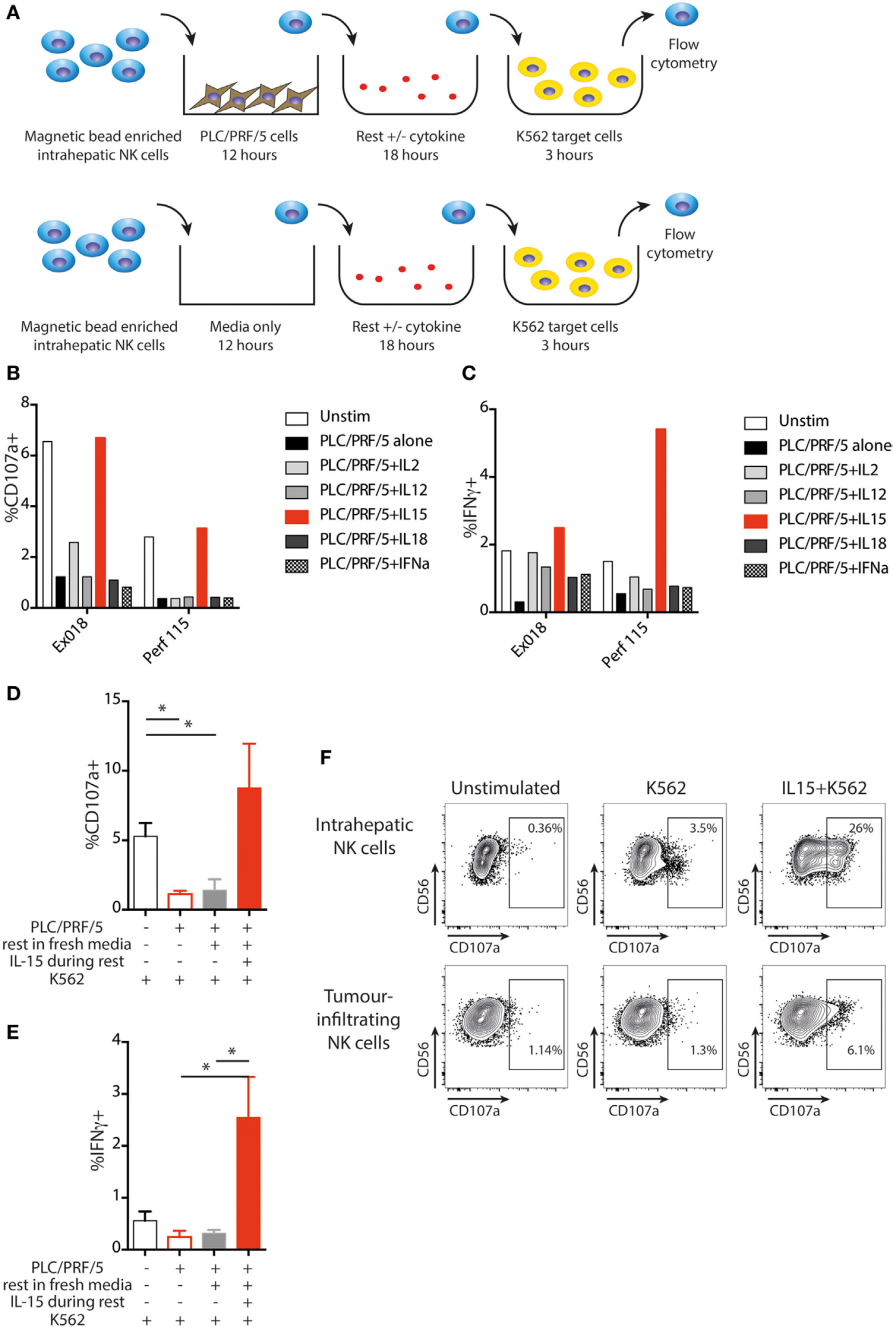
IL-15 restores NK cell function following PLC/PRF/5 coculture. **(A)** Diagram showing experimental design of PLC/PRF/5 coculture followed by rest in fresh media ± cytokine before K562 challenge. **(B)** CD107a staining following K562 challenge after PLC/PRF/5 coculture, then rest overnight in fresh media ± the indicated cytokine using intrahepatic NK cells isolated from a liver explant (Ex) and from a liver perfusate (Perf). **(C)** Intracellular IFNγ staining following K562 challenge after PLC/PRF/5 coculture, then rest overnight in fresh media ± the indicated cytokine using intrahepatic NK cells isolated from a liver explant (Ex) and from a liver perfusate (Perf). **(D)** Summary data showing CD107a staining following K562 challenge of intrahepatic NK cells unstimulated, after PLC/PRF/5 coculture, after PLC/PRF/5 coculture then rest in fresh media and after PLC/PRF/5 coculture then rest in IL-15 containing media (*n* = 5 liver samples). **(E)** Summary data showing intracellular IFNγ staining following K562 challenge of intrahepatic NK cells unstimulated, after PLC/PRF/5 coculture, after PLC/PRF/5 coculture then rest in fresh media and after PLC/PRF/5 coculture then rest in IL-15 containing media (*n* = 5 liver samples). **(F)** Example of CD107a staining on paired intrahepatic and tumor-infiltrating NK cells following K562 challenge after overnight rest with IL-15. Groups were compared using unpaired *t* test with Welch’s correction. *p* ≤ 0.05 was considered to be significant for all tests. Figures are labeled: *p ≤ 0.05.

Next, we sought to investigate mechanisms by which intratumoral NK cell function might be restored. NK cells were removed from overnight PLC/PRF/5 coculture and rested in fresh medium overnight in the presence of different cytokines, followed by challenge with K562 cells as before (experimental approach in Figure 7A). Degranulation was improved by overnight treatment with IL-15 but not IL-2, IL-12, or IL-18, compared to medium alone, before K562 challenge (Figure 7B). In the same experiments, IFNγ production was partially recovered by all the cytokines tested, but IL-15 showed the most pronounced boosting (Figure 7C). Using NK cells extracted from five different livers, we observed that NK cell degranulation was significantly impaired following incubation with PLC/PRF/5, even after a further 18 h resting in media, but was consistently restored to baseline levels after incubation with IL-15 (Figure 7D). NKG2D expression was increased following IL-15 stimulation, but recovery of PLC/PRF/5-induced NKG2D downregulation was modest (Figure S4A in Supplementary Material). NK cell IFNγ was also consistently boosted to even higher levels than those seen without exposure to PLC/PRF/5 after overnight incubation with IL-15 rather than media alone (Figure 7E). Both liver-resident and non-resident NK cells recovered function in a similar way to the global population following IL-15, although there was a suggestion that the liver-resident NK cells recovered cytotoxic function more while non-resident NK cells recovered more cytokine production (Figure S4 in Supplementary Material).

Finally, we tested the capacity of IL-15 to rescue NK cells after *in vivo* exposure to HCC. Intrahepatic and intratumoral lymphocytes from the same liver were incubated with IL-15 or media alone overnight and then challenged with K562. NK cells isolated from the liver showed a clear degranulation response to K562 cells that was strikingly augmented by IL15 (Figure 7F). By contrast, NK cells extracted from the HCC were unable to respond to K562 cells (CD107a comparable to baseline) unless they had been pre-stimulated with IL-15. IL-15 boosted degranulation of tumor-infiltrating NK cells to a higher percentage than the basal level seen upon K562 challenge of NK cells from the unaffected distant liver margin (Figure 7F).

## Discussion

Here, we use direct *ex vivo* analysis of freshly isolated human tumor and liver lymphocytes to show that HCC and CRC metastases are populated by high frequencies of NK cells with liver-resident and liver-infiltrating phenotypes. We find that NKG2D expression is strikingly enriched on the CXCR6^+^ liver-resident NK cell subset but is consistently downregulated on both these and their non-resident counterparts in the setting of liver tumors, with impaired proliferation of the NKG2D^lo^ fraction. Intratumoral NK cells have low cytolytic and non-cytolytic potential, even more so than the tissue-resident subset of the uninvolved liver. These features are recapitulated when liver NK cells have direct contact with an HCC cell line and are amenable to reversal by IL-15.

T cells are generally considered to make up the majority of the lymphocytic infiltrate in liver tumors, although only a small fraction of these are likely to be tumor-specific (3). However, our data reveal that NK cells are more prevalent in liver tumors than generally recognized, tending to outnumber CD8 T cells in both HCC and CRC metastases. This is in contrast to primary colorectal tumors, where few NK cells are found (27), with this difference being underscored by our analysis of primary colon and secondary liver metastases resected simultaneously from the same patient. These data suggest that tumors can co-opt the predominant immune infiltrates of the organ they metastasize to, rather than imposing an infiltrate dictated by the primary cancer. We show for the first time that HCC and CRC metastases contain the recently defined CXCR6^+^ human liver-resident NK cell subset as well as NK cells with a liver-infiltrating (non-resident) phenotype. Previous work by our group and colleagues has shown that these cells are NK cells as evidenced by their expression of CD56 and Eomes (20, 28) and are transcriptionally distinct from ILC1 cells (21). It is possible that NK cells residing in the liver move into tumors or that non-resident NK cells acquire a resident phenotype on encountering relevant signals within the tumor.

Unlike T cells, NK cells are capable of providing tumor surveillance and tumor immunity without specificity for tumor antigen or neoantigen, thereby offering a large pool of potential effectors (5). The relevance of assessing NK cell responses in liver tumor immunity has been supported by clinical studies finding associations between increased peripheral or tumor-infiltrating NK cell frequencies and better survival with HCC (29) and CRC metastases (30), respectively. A similar large clinical series would be required to assess whether the variable proportion of tissue-resident NK cells infiltrating liver tumors correlates with prognosis, as recently reported for lung-resident T cells (23, 24). One potential advantage conferred by liver-resident NK cells is the strikingly high expression of NKG2D we observed, which should facilitate sensing of NKG2D-L on tumors. Our *ex vivo* data show that NKG2D expression remains significantly higher on the CXCR6^+^ tissue-resident fraction of NK cells within HCC and CRC metastases, even though it is lower than on their counterparts in unaffected distant liver margins. *In vitro* exposure to an HCC cell line recapitulated the *ex vivo* finding that the tumor-induced downregulation and internalization of NKG2D is largely attributable to reductions on the tissue-resident fraction of NK cells. The selective reduction of NK cell Ki67 expression on the NKG2D^lo^ fraction suggests that NKG2D downregulation impedes their proliferative renewal within the tumor milieu.

By comparing intratumoral and matched unaffected liver NK cell function according to tissue-residency, we were able to determine that the majority of the functional impairment found within tumors is attributable to the hypo-functionality of liver-resident NK cells. Thus the overall reduction in NK cell granzyme B within liver tumors is primarily driven by the low levels in the liver-resident fraction, with the non-resident cells maintaining much higher expression. IFNγ production by peripheral NK cells from HCC patients in response to IL-12 and IL-18 was in keeping with that of another HCC cohort (31) and by tumor-infiltrating NKs was similar to that seen in NK cells infiltrating breast tumors (32). We did not investigate the expression of IL12 or IL18 receptor but others have shown that IL12Rβ1 expression falls and IL12Rβ2 expression is increased in CXCR6^+^ NK cells in the liver, suggesting subtle tuning of cytokine responses (33). Taken together, our data show that both cytolytic and non-cytolytic (IFNγ) antitumor effector functions are further impaired within liver tumors compared to NK cells from uninvolved liver. Such functional paralysis is likely due to a combination of factors, with previous studies implicating regulatory T cells (31) and myeloid-derived suppressor cells (34) infiltrating HCC. Our experiments implicate a direct contribution from HCC cells, that can drive NKG2D internalization and recapitulate the defects in production of cytotoxic and cytokine mediators in a contact-dependent manner, without the need for accessory cells.

*In vitro* blockade did not confirm a clear role for NKG2D downregulation or TGFβ in the HCC-mediated functional inhibition of NK cells we observed. Likewise, overnight rest in fresh media did not restore tumor NK cell degranulation or IFNγ production, suggesting that functional impairment was not due to a reversible metabolic restriction or competition for nutrients in coculture. By contrast, IL-15 was able to recover cytotoxic capacity and also conferred the most efficient boosting of IFNγ production. This enhancement in degranulation and IFNγ production was consistent, recovering function even when coculture had rendered the NK cells almost totally anergic. The use of IL-15 as an immunotherapeutic approach in HCC is supported by data from mouse models. An NK cell line transfected with IL-15 signals in an autocrine fashion to control HCC in mouse models (35). K562 cells transfected with membrane bound IL-15 and 4-1BB ligand can expand and activate NK cells for use in HCC immunotherapy (36). IL-15 is being used to maintain *ex vivo* activated NK cells for infusion in the context of hematological malignancy and solid tumors in clinical trials (NCT01875601, NCT01385423) (37). Similar approaches with modified IL-15/IL-15Rα complexes (IL-15 superantigen) are being developed for use in clinical trials (38). However, administration of IL-15 and associated molecules can cause significant toxicity (39), which appears to be related to systemic production of IFNγ by NK cells (40). To overcome this, IL-15 could be targeted to cancer cells by fusion with specific antibodies, shown to enhance antitumor activity in animal models (41). Our example of *ex vivo* tumor-infiltrating NK cells being re-activated by IL-15 suggests that direct delivery could be used in liver tumors to activate local NK cells *in vivo* while minimizing systemic toxicity.

In conclusion, human liver tumors have high frequencies of NK cells, a large fraction of which have the features of a recently described liver-resident subset. We find that tissue-resident NK cells have increased expression of NKG2D compared to their non-resident counterparts but remain susceptible to downregulation of this key receptor in tumors. The functional paralysis of intratumoral NK cells partly reflects features of NK cells residing in the tolerogenic liver environment, with additional inhibition imposed by the tumor. IL-15 is able to boost functionality of intrahepatic NK cells following exposure to HCC, underscoring the therapeutic potential of this cytokine to harness the potent antitumor potential of this large component of liver tumor infiltrates.

## Ethics Statement

This study was carried out in accordance with the recommendations of University College London-Royal Free Hospital Research Ethics Committee and the South East Coast Research Ethics Committee. The protocol was approved by the University College London-Royal Free Hospital Research Ethics Committee and the South East Coast Research Ethics Committee. All subjects gave written informed consent in accordance with the Declaration of Helsinki.

## Author Contributions

NE and MM prepared the manuscript and designed the study. NE, LS, KS, LP, AB, DO, NS, and W-CH performed experiments. NE analyzed the data. GF and BD provided tissue samples. All authors reviewed the manuscript.

## Conflict of Interest Statement

The authors declare that the research was conducted in the absence of any commercial or financial relationships that could be construed as a potential conflict of interest.
