# Supplementary figures and images for: IL-15 Overcomes Hepatocellular Carcinoma-Induced NK Cell Dysfunction
Source: Front Immunol. 2018 May 9;9:1009. doi: 10.3389/fimmu.2018.01009 (PMC5954038; doi:10.3389/fimmu.2018.01009)

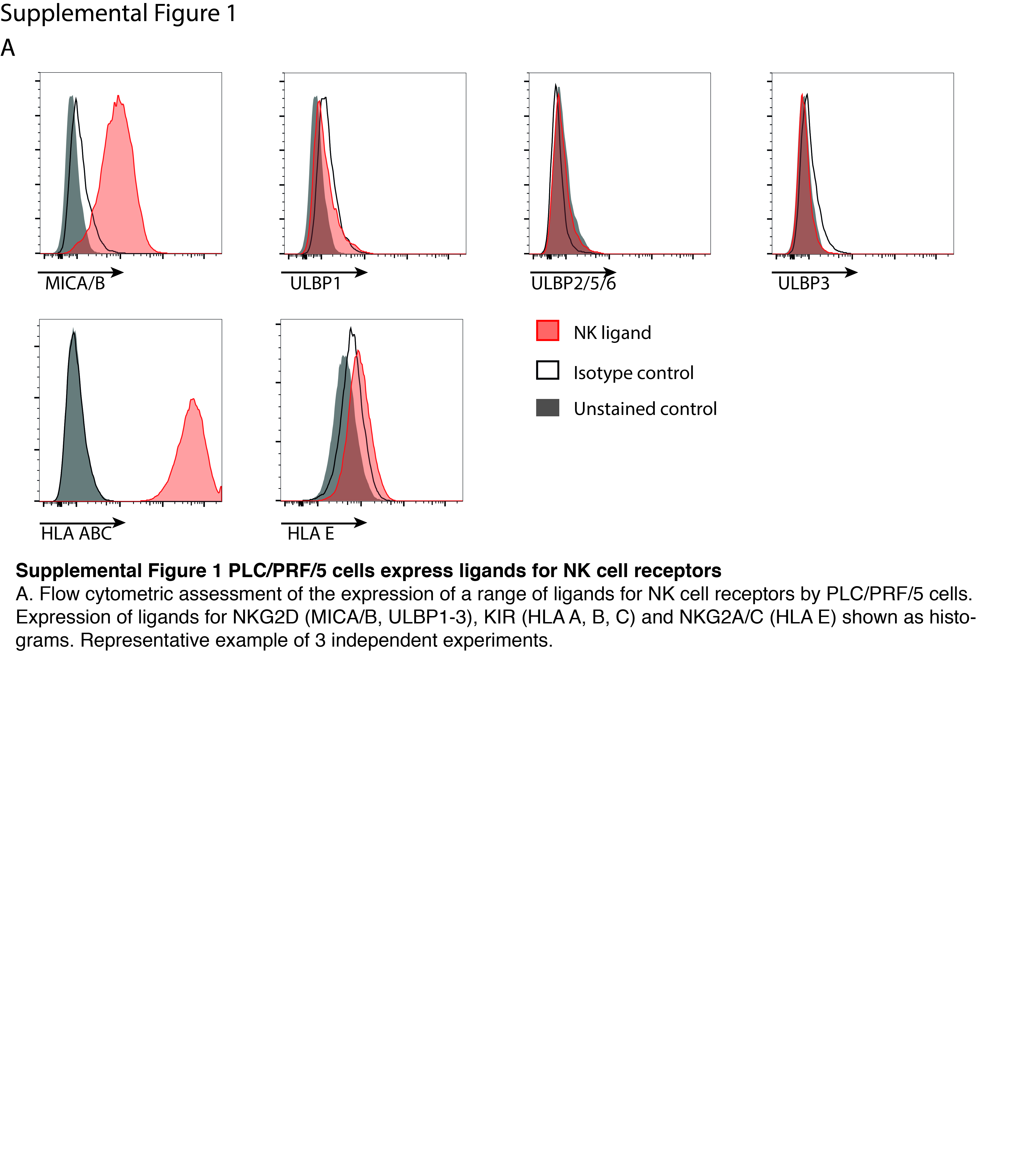

Supplement: Supplementary file 1 [file Image_1.jpeg]

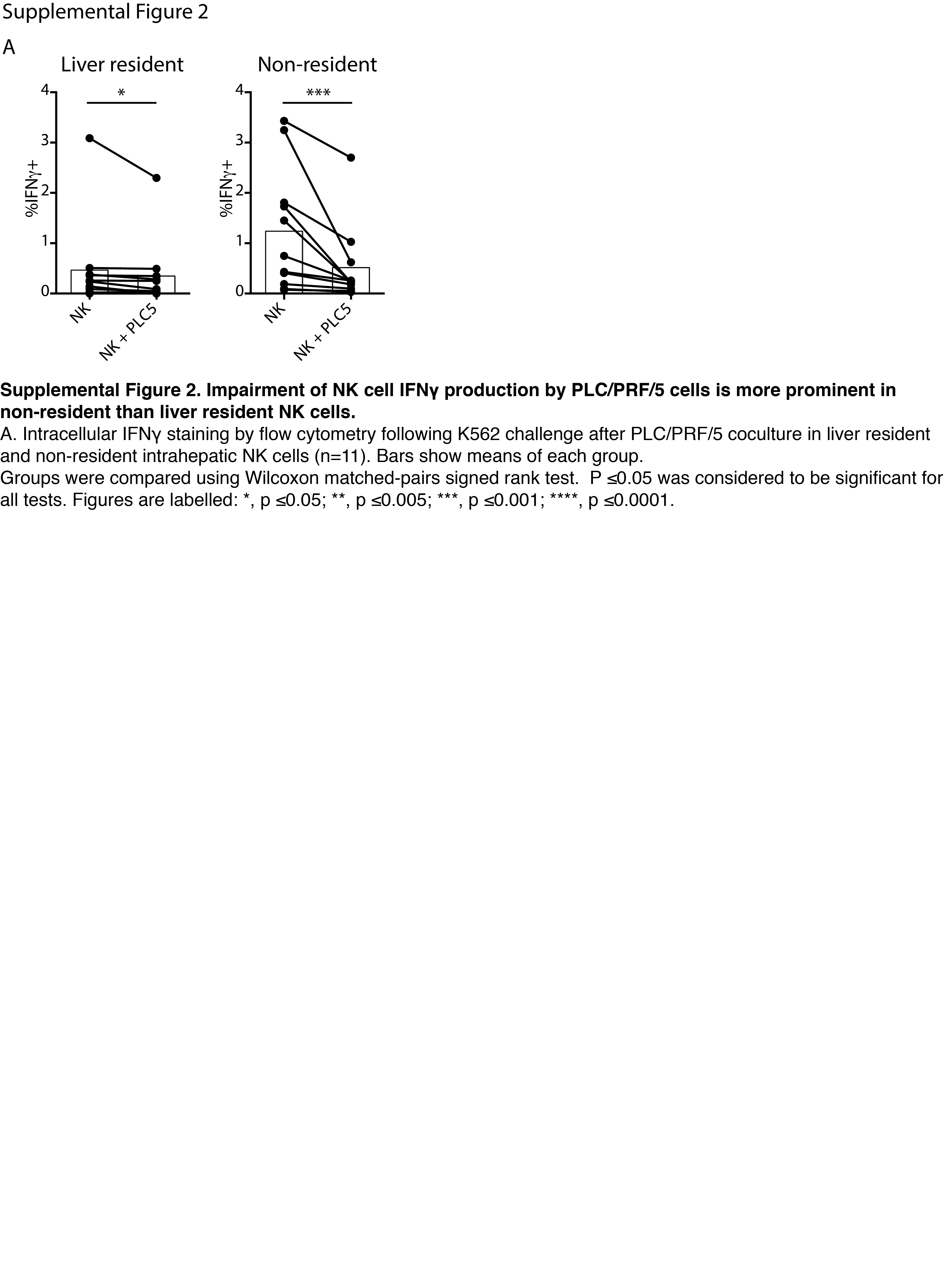

Supplement: Supplementary file 2 [file Image_2.jpeg]

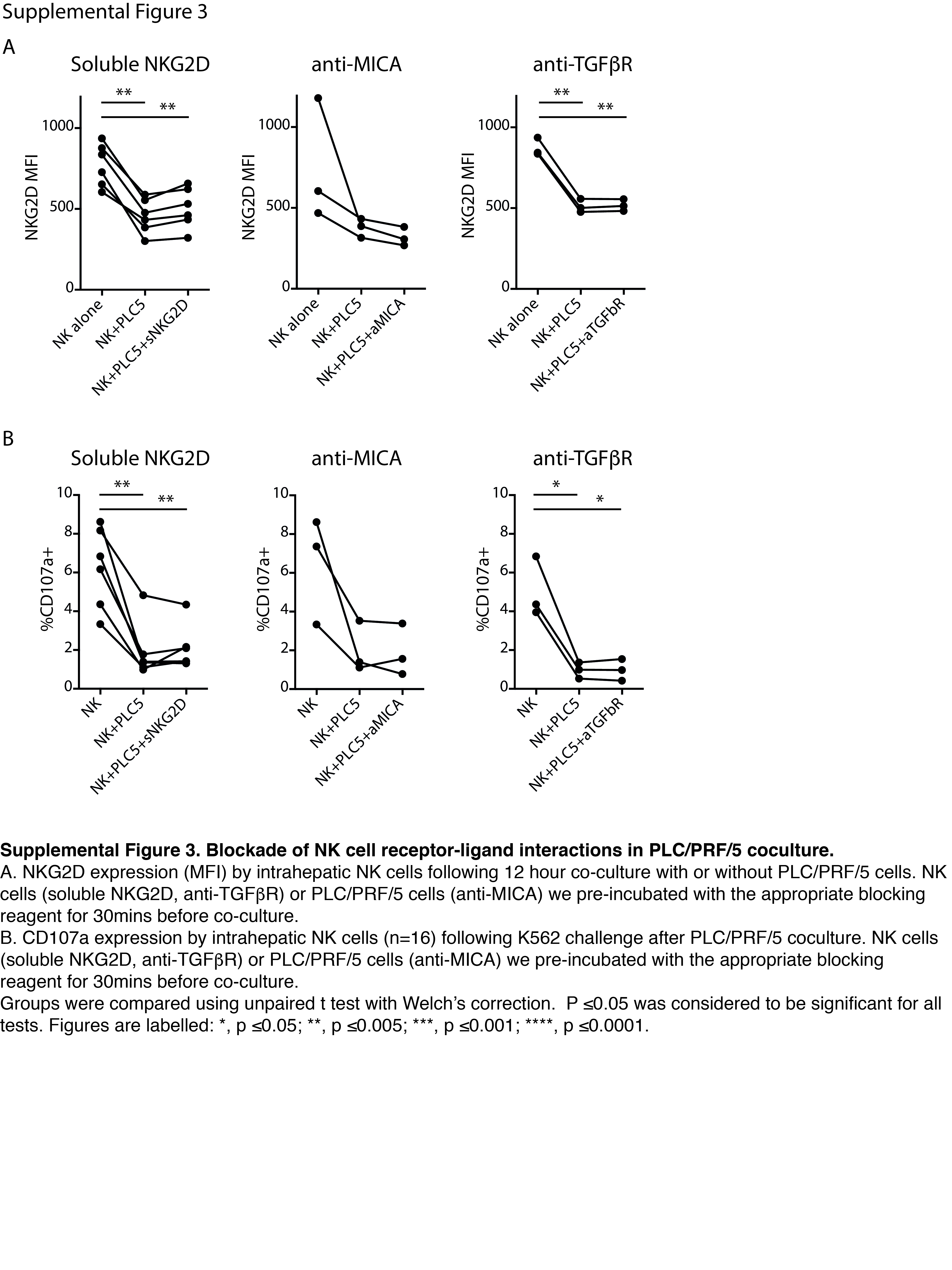

Supplement: Supplementary file 3 [file Image_3.jpeg]

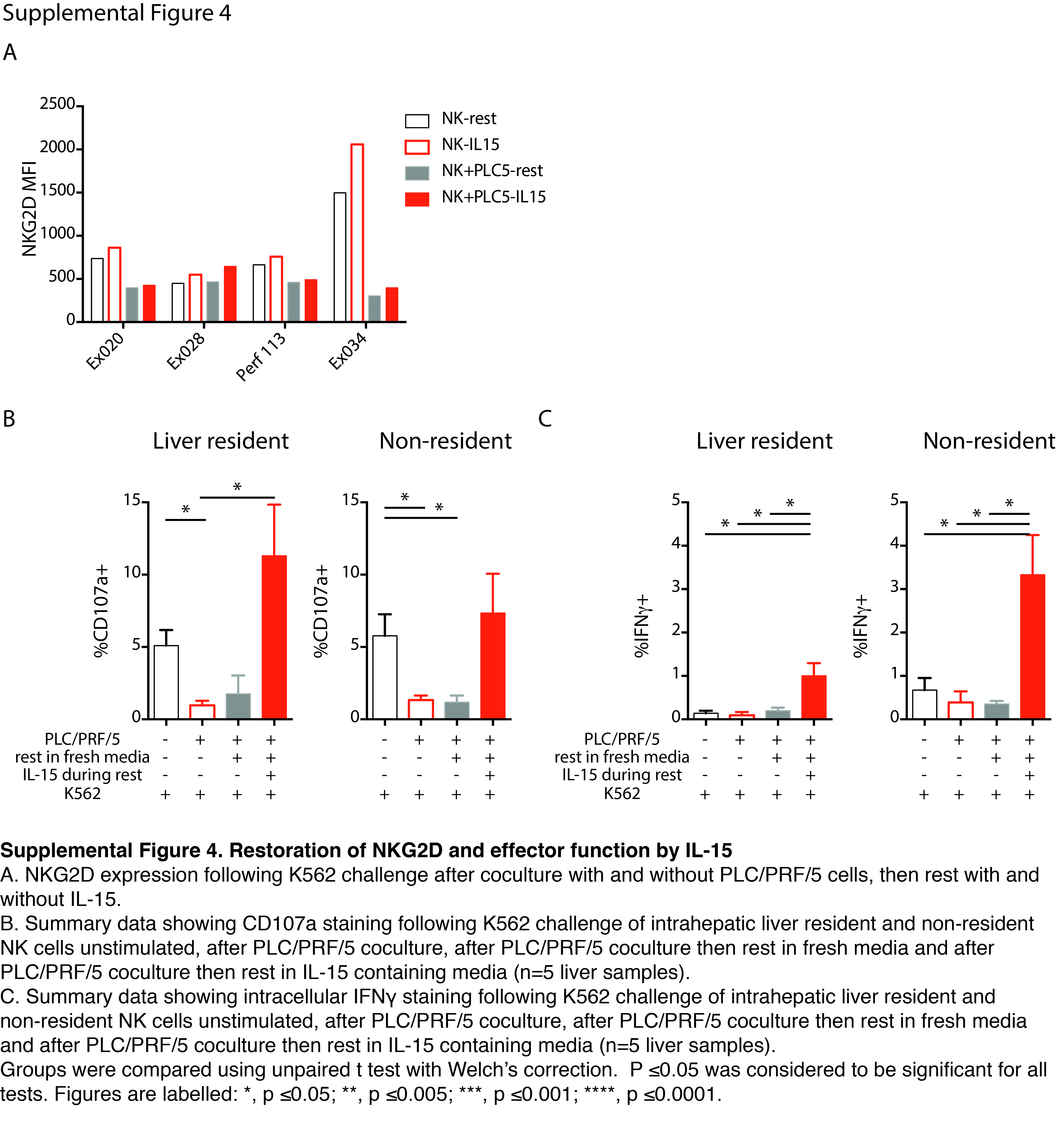

Supplement: Supplementary file 4 [file Image_4.jpeg]
